# Delirium and Anxiety Outcomes Related to Visiting Policy Changes in the Intensive Care Unit During the COVID-19 Pandemic

**DOI:** 10.3389/fnagi.2022.845105

**Published:** 2022-03-02

**Authors:** Bomi Kim, Jaehwa Cho, Jin Young Park, Hesun Erin Kim, Jooyoung Oh

**Affiliations:** ^1^Institute of Behavioral Sciences in Medicine, Yonsei University College of Medicine, Seoul, South Korea; ^2^Department of Pulmonary and Critical Care Medicine, Gangnam Severance Hospital, Yonsei University College of Medicine, Seoul, South Korea; ^3^Department of Psychiatry, Yongin Severance Hospital, Yonsei University College of Medicine, Yongin, South Korea; ^4^Department of Psychiatry, Gangnam Severance Hospital, Yonsei University College of Medicine, Seoul, South Korea

**Keywords:** COVID-19, intensive care unit, delirium, anxiety, family visit, motor subtype

## Abstract

**Objective:**

To evaluate the effect of intensive care unit (ICU) visit on the incidence of delirium, delirium subtype, and anxiety level in ICU patients.

**Methods:**

Trained psychiatrists and nurses evaluated ICU patients for delirium, delirium subtypes, and anxiety. Propensity score matching (PSM) was used to retrospectively analyze the data. Then, we compared the differences in the incidence of delirium, delirium subtypes, and anxiety level before and after the ICU visit ban. Logistic regression was conducted to identify the risk factors for delirium subtypes and high anxiety levels.

**Results:**

After PSM, there was no statistically significant difference in the incidence of delirium between the non-visiting and restrictive visiting groups (non-visiting 27.4% versus restrictive visiting 30.9%, *p* = 0.162). The proportion of hyperactive and mixed subtypes was higher in the non-visiting than in the restrictive visiting group (non-visiting 35.3 and 30.1% versus restrictive visiting 27.7 and 20.1%, *p* = 0.002). The anxiety level was higher in the non-visiting than in the restrictive visiting group (state-trait anxiety inventory score: non-visiting 53.46 ± 4.58 versus restrictive visiting 52.22 ± 6.50, *p* = 0.009). Patients who stayed in the ICU during the visit ban were more likely to have hyperactive (*p* = 0.005) and mixed subtype (*p* = 0.001) than those who did not. Moreover, patients who stayed in the ICU during the visit ban were more likely to experience high anxiety levels than those who did not (*p* < 0.001).

**Conclusion:**

Prohibition of ICU visits during COVID-19 pandemic did not affect the incidence of delirium during COVID-19 but could change the delirium subtype and raise anxiety level. Moreover, visiting prohibition was a risk factor for non-hypoactive delirium subtype and high anxiety levels. Therefore, ICU visits are important in dealing with delirium subtypes and anxiety in ICU patients.

## Introduction

Delirium is a syndrome characterized by acute and fluctuating cognitive impairments, which include the clouding of consciousness, inattention, and disorientation. The prevalence of delirium is high in older hospitalized patients, mechanically ventilated patients, or critically ill patients (Ely et al., 2001a; van den Boogaard et al., 2012; Mehta et al., 2015). It is known that delirium is associated with adverse effects such as longer intensive care unit (ICU) stays, higher hospital costs, prolonged mechanical ventilation, cognitive decline after ICU discharge, and increased morbidity and mortality (Ely et al., 2001a; Ouimet et al., 2007; Pisani et al., 2009; Mehta et al., 2015; Goldberg et al., 2020). Recently, it has been reported that the incidence of delirium in critically ill patients with COVID-19 is very high, reaching more than 50% (Pun et al., 2021; Williamson et al., 2022). In particular, delirium is also known as a strong predictor of mortality in hospitalized COVID-19 patients (Fernández-Jiménez et al., 2021).

Although it is well known that delirium is associated with negative clinical outcomes, the severity and prognosis of delirium vary depending on three motoric subtypes: hyperactive (agitation, hallucinations, aggression), hypoactive (sedation, inattentiveness, lethargy, motor slowness), and mixed subtype (fluctuation between hyperactive and hypoactive subtypes). Prior studies have shown that patients with the hypoactive subtype have a more severe prognosis than those with the hyperactive and mixed subtypes (Yang et al., 2009; Krewulak et al., 2018). Patients with the hyperactive subtype have less severe outcomes than those with the other subtypes but might give rise to caring problems or inconvenience to caregivers (van den Boogaard et al., 2012; Krewulak et al., 2018).

Anxiety is also common psychiatric symptom experienced by patients in ICUs. Several studies have reported that more than half of ICU patients showed moderate or severe anxiety levels (McKinley et al., 2004; Li and Puntillo, 2006). ICU patients might experience anxiety, including fear of extreme pain or the treatment process itself, including fright caused by unfamiliar medical devices, and concern about isolation from the family (Lee and Kang, 2011). In line with this, a previous study pointed out that anxiety felt by ICU patients was divided into physical, environmental, and interpersonal anxiety. Among them, the biggest cause of interpersonal anxiety was isolation from family members (Han and Park, 2002).

For early and proper intervention of delirium and anxiety in ICU patients, pharmacological interventions are mainly conducted. In terms of pharmacological interventions for delirium, previous evidence mainly focused on patients with sleep problems or behavioral problems (Peritogiannis et al., 2009; Flannery et al., 2016), and antipsychotic medications have not been effective in changing the course of delirium (Page et al., 2013; Girard et al., 2018). Furthermore, there may be several side effects of the antipsychotics (Devlin et al., 2018). On the other hand, medications such as sedatives are used to alleviate the patient’s anxiety and agitation in ICU, but overuse of the medications is related to potential risks such as increased length of stay (Jacobi et al., 2002; Tracy and Chlan, 2011). Therefore, clinical practice guidelines have suggested nonpharmacological interventions for delirium and anxiety, one of which is family presence and participation (Rosa et al., 2017; Pun et al., 2019).

After the COVID-19 pandemic has rapidly spread since its outbreak at the end of 2019, almost all hospitals have enforced policies to ban ICU visits to prevent infection worldwide. Previous studies have reported that a flexible visit policy in the ICU can lower the incidence of delirium and is associated with lower level of anxiety than a restricted visit policy (Fumagalli et al., 2006; Junior et al., 2018). Although the association between the role of family visits and delirium subtype has not been examined enough, a previous study found that simulated family presence had reduced agitation in patients with hyperactive or mixed delirium (Waszynski et al., 2018). Based on this evidence, it can be expected that the number of patients with hyperactive or mixed subtype might have increased from the time of the visit ban in ICU, during COVID-19 pandemic. In line with this, no visiting policy might negatively affect the prognosis of delirium and anxiety in ICU patients, but the association between no visiting policy and mental illness including delirium and anxiety during the COVID-19 pandemic has not been reported yet.

The current study aimed to examine the possible effect of family visits on the incidence of delirium, the subtype of delirium, and the level of anxiety in ICU patients. In other words, we assessed the influence of changes in visiting policy after the COVID-19 pandemic on delirium incidences, motor subtypes of delirious patients, and anxiety levels of non-delirious patients in a clinical cohort of a university hospital using data from January 2019 to May 2021. We hypothesized that the incidence of overall delirium, the rates of hyperactive and mixed subtypes, and the level of anxiety would increase, and that the rates of hypoactive subtype would decrease during the visit prohibition period (i.e., non-visiting) in the ICU compared to the period in which the previous usual visiting rules (i.e., restrictive visiting) were applied.

## Materials and Methods

### Study Design and Participants

This study was conducted as retrospective observational study used data from patients who stayed in the ICU of Gangnam Severance Hospital from January 1, 2019, to May 3, 2021. Restrictive family visits (“restrictive visiting”) implemented once or twice a day from January 1, 2019, to August 24, 2020, and family visits were completely banned (“non-visiting”) due to the spread of the COVID-19 pandemic from August 25, 2020, to May 3, 2021. There were no other major changes in ICU operating conditions between the two periods, except for visiting policies. As a part of the ICU Distress and Delirium Management project at the hospital (Oh et al., 2015), trained nurses screened delirium using the Confusion Assessment Method for the Intensive Care Unit (CAM-ICU) for ICU patients three times a day (Ely et al., 2001b; Heo et al., 2011). Every day at 10 a.m., based on the evaluation records of nurses and the current status of patients, psychiatrists evaluated the patient’s delirium status and classified it into three groups: comatose, delirious, and non-delirious. Delirious groups were evaluated for delirium subtype, and non-delirious groups were evaluated for pain and anxiety.

**FIGURE 1:**
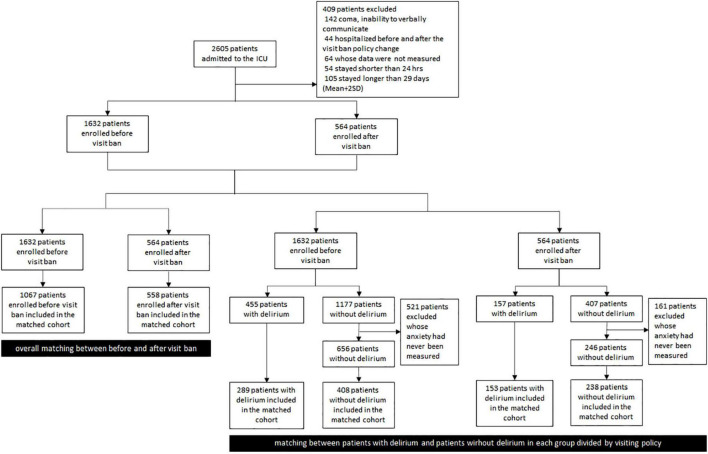
A diagram on the patient enrollment and matching process.

A total of 2,605 patients stayed in the ICU from January 1, 2019, to May 3, 2021 (see Figure 1). We excluded 142 patients who were continuously comatose or unable to communicate, 44 patients who stayed both before and after the visit ban, 64 patients whose one or more indicators such as restraint use, intubation use, and Acute Physiology and Chronic Health Evaluation II (APACHE II) were not measured or recorded, and 54 patients who stayed in the ICU for less than 24 h. A total of 105 patients who had extreme values with an ICU stay period of more than 2 standard deviations higher than the average were also excluded. If delirium was experienced at least once during ICU stays, the patients were classified as “delirious patients” group. COVID-19 patients were not included because they were treated in an isolation ward. The Institutional Review Board of Gangnam Severance Hospital approved the study.

### Assessment

#### Diagnosis of Delirium

Patients were diagnosed with delirium if there had been delirium at least once in the CAM-ICU. The CAM-ICU is a commonly used tool for screening delirium in two steps (Ely et al., 2001b). Step 1 is the assessment of sedation status using the Richmond Agitation-Sedation Scale (RASS). The score consists of −5 (unconscious) to +4 (aggressive), and if the score is higher than −3, the delirium state is evaluated with the CAM-ICU in the next step. Delirium state judgment is conducted for the following four areas: acute mental state change (feature 1), attention deficit (feature 2), consciousness level change (feature 3), and unsystematic thinking (feature 4). Among the patients who satisfy both features 1 and 2, if they show features 3 or 4, the patients are assessed as having delirium (Guenther et al., 2010).

#### Delirium Subtype

Delirium Motor Subtype Scale (DMSS) and RASS were used to measure delirium subtypes, which were divided into hyperactive, hypoactive, mixed, and no subtypes according to mental motor disorders of delirium patients (Kim et al., 2018). DMSS consists of a total of 11 motor items, 4 items related to hyperactive subtype, and 7 items related to hypoactive subtype. If it falls under two or more items among quantitative increases in exercise activities, loss of activity control, restlessness, and wandering, the patients were classified as the hyperactive subtype. On the other hand, the patients were classified as the hypoactive subtype if they had two or more items, such as the quantitative reduction of activity, decreased behavioral speed, decreased awareness of the surroundings, decreased quantitative speech, decreased speech speed, indifference, and withdrawn consciousness. If the patients had both subtypes, they were classified as mixed subtypes, and if not, they were classified as no type (van den Boogaard et al., 2012; Kim et al., 2018). In this study, no patients were classified as no type. Some patients with missing DMSS values were evaluated for delirium subtypes using RASS instead of DMSS. In the case of RASS, patients who tested positive for delirium were classified as hypoactive if they were between −3 and 0, hyperactive type if they exceeded 0, and mixed type if RASS scores were included in both categories during the same day (Ely et al., 2003; van den Boogaard et al., 2012).

#### Anxiety

Anxiety was measured using the six-item short-form State-Trait Anxiety Inventory (STAI-6) (Marteau and Bekker, 1992). This scale is composed of states with a short form of 6-item by adapting the original 20-item state scale and has high internal reliability and validity (McDermott et al., 2013). The STAI-6 includes emotional states such as relaxed, upset, worried, and asks about the frequency of experiencing the states. A 4-point scale was used to measure anxiety, and the original total scores ranged from 6 to 24. However, to compare with the score from the full 20-item STAI, the range of scores was converted from 20 to 80 (Hou et al., 2015).

### Data Collection

We obtained demographic and clinical information including age, sex, ICU stay, type of admission (medical/surgical), surgery at admission, and operation status (none/elective/emergency) from electronic medical records (EMR). We also collected the APACHE II score when the patients were admitted to the ICU to estimate the severity of the disease. Data on intubation, restraint, and ventilator use were reviewed, and if the patients had used the intubation, restraint, and ventilator at least once during ICU stay, they were considered to have been used. Records of analgesics (tramadol, oxycodone, pethidine, fentanyl, morphine, acetaminophen, codeine phosphate hydrate, and ibuprofen) and sedatives (midazolam, remifentanil, lorazepam, propofol, fentanyl, and dexmedetomidine hydrochloride) were also obtained from the EMR. As outcome measures, the incidence of delirium, delirium subtype, and anxiety level were collected.

### Statistical Analysis

The propensity score matching (PSM) method was implemented to reduce selection bias and balance characteristics between the two groups because of the non-randomized assignment of patients. Before PSM, the differences in characteristics between the restrictive visiting group and the non-visiting group were analyzed using the chi-square test for categorical variables and independent *t*-test for continuous variables. Some clinical variables showing significant differences between the groups such as restraint use were used as covariates to calculate propensity scores. Propensity scores were calculated by logistic regression analysis using clinical characteristics, which can act as confounders. The prediction variables to calculate the propensity score were different among the three groups: all patients, delirious patients, and non-delirious patients. ICU stay, type of admission, surgery at admission, operation status, APACHE II score, intubation use, restraint use, ventilator use, analgesic medication use, and sedative medication use were used to calculate the propensity scores for all patients. Restraint use, ventilator use, and APACHE II were used to obtain the scores for delirious patients, and then, restraint use, intubation use, ventilator use, APACHE II, and sedative medication use were used for non-delirious patients. The calculated propensity scores were matched between similar individuals to compare the incidence of delirium, delirium subtype, and the anxiety level of the two groups, the restrictive visiting group and the non-visiting group. The estimation algorithm was a logistic regression model, and the matching algorithm was 1:2 nearest neighbor matching with no replacement. The 1:2 PSM (1 after visit ban and 2 before visit ban patients) was conducted on each of the three groups: all patients, delirious patients, and non-delirious patients. All absolute standardized mean differences were less than 0.1. We included 1,625 patients in the analysis for the incidence of delirium, 558 delirious patients in the analysis for the delirium subtype, and 646 non-delirious patients in the analysis for the level of anxiety. The differences in characteristics between the two matched groups were analyzed using the chi-square test for categorical variables and independent *t*-test for continuous variables. Then, a multinomial logistic regression model was used to identify risk factors for delirium subtypes [hypoactive (the referent group), hyperactive, mixed] in all patients before and after visit ban. In this analysis, we utilized the variables as predictors that showed significant differences between the groups before matching including visiting status. Finally, patients in the matched cohort were divided into the high anxiety group (higher than the average) and low anxiety group (lower than the average), and a binary logistic regression model was used to analyze independent predictors of anxiety. Statistical analyses were performed using the R software 4.1.1.

## Results

### Demographic and Clinical Characteristics Before and After Propensity Score Matching

The demographic and clinical characteristics before and after PSM are presented in Table 1. Before PSM, among a total of 2,196 patients, 564 stayed in the ICU after the visit ban. APACHE II, intubation use, restraint use, ventilator use, and sedative medication use were significantly higher in patients after the visit ban than those before the visit ban. 455 patients were in the restrictive visiting group and 157 patients were in the non-visiting group among 612 delirious patients. APACHE II, restraint use, and ventilator use were significantly higher in delirious patients after the visit ban than those before the visit ban, and there was no difference in the others. As for the non-delirious group, 656 patients were in the restrictive visiting group and 246 patients were in the non-visiting group among 902 patients, and APACHE II, intubation use, restraint use, ventilator use, and sedative medication use were significantly higher in non-delirious patients after the visit ban than those before the ban.

**TABLE 1:** Demographics and clinical characteristics before and after matching.

Variables	Categories	Before matching			After matching		
		Restrictive visiting	Non-visiting	*p*	Restrictive visiting	Non- visiting	*p*
All patients		(*N* = 1632)	(*N* = 564)		(*N* = 1047)	(*N* = 559)	
Age, mean (SD)		66.2 (16.6)	65.7 (16.0)	0.574	66.1 (16.7)	65.6 (16.0)	0.606
Male, No. (%)		990 (60.7)	362 (64.2)	0.152	633 (60.5)	358 (64.0)	0.176
ICU days, mean (SD)		3.9 (4.6)	4.1 (4.8)	0.509	4.53 (5.0)	4.06 (4.8)	0.064
Type of admission, No. (%)	Medical	422 (25.9)	182 (32.3)	0.004	317 (30.3)	180 (32.2)	0.461
	Surgical	1210 (74.1)	382 (67.7)		730 (69.7)	379 (67.8)	
Surgery at admission, No. (%)		1162 (71.2)	360 (63.8)	0.001	690 (65.9)	358 (64.0)	0.490
Operation status, No. (%)	None	569 (34.9)	227 (40.2)	0.007	409 (39.1)	225 (40.3)	0.285
	Elective	776 (47.5)	225 (39.9)		456 (43.6)	223 (39.9)	
	Emergency	287 (17.6)	112 (19.9)		182 (17.4)	111 (19.9)	
APACHE II score, mean (SD)		18.2 (8.3)	20.4 (8.7)	<0.001	19.71 (8.6)	20.3 (8.6)	0.221
Intubation use, No. (%)		702 (43.0)	292 (51.8)	<0.001	541 (51.7)	291 (52.1)	0.924
Restraint use, No. (%)		831 (50.9)	350 (62.1)	<0.001	623 (59.5)	345 (61.7)	0.418
Ventilator use, No. (%)		665 (40.7)	304 (53.9)	<0.001	544 (52.0)	299 (53.5)	0.594
Analgesics use, No. (%)		1222 (74.9)	396 (70.2)	0.035	736 (70.3)	396 (70.8)	0.865
Sedatives use, No. (%)		478 (29.3)	195 (34.6)	0.022	359 (34.3)	195 (34.9)	0.854

**Delirious patients**		**(*N* = 455)**	**(*N* = 157)**		**(*N* = 289)**	**(*N* = 153)**	

Age, mean (SD)		71.8 (15.9)	70.5 (16.1)	0.398	72.4 (14.9)	70.4 (16.2)	0.175
Male, No. (%)		272 (59.8)	99 (63.1)	0.529	167 (57.8)	96 (62.8)	0.364
ICU days, mean (SD)		7.3 (6.0)	7.6 (6.5)	0.569	7.68 (6.1)	7.34 (6.2)	0.577
Type of admission, No. (%)	Medical	168 (36.9)	67 (42.7)	0.237	101 (35.0)	64 (41.8)	0.187
	Surgical	287 (63.1)	90 (57.3)		188 (65.1)	89 (58.2)	
Surgery at admission, No. (%)		284 (62.4)	88 (56.1)	0.189	185 (64.0)	87 (56.9)	0.171
Operation status, No. (%)	None	212 (46.6)	85 (54.1)	0.263	130 (45.0)	81 (52.9)	0.268
	Elective	140 (30.8)	41 (26.1)		87 (30.1)	41 (26.8)	
	Emergency	103 (22.6)	31 (19.7)		72 (24.9)	31 (20.3)	
APACHE II score, mean (SD)		22.0 (8.1)	23.5 (8.6)	0.048	23.2 (8.6)	23.5 (8.4)	0.705
Intubation use, No. (%)		280 (61.5)	100 (63.7)	0.700	206 (71.3)	97 (63.4)	0.112
Restraint use, No. (%)		391 (85.9)	148 (94.3)	0.008	277 (95.9)	145 (94.8)	0.781
Ventilator use, No. (%)		237 (52.1)	102 (65.0)	0.007	183 (63.3)	98 (64.1)	0.962
Analgesics use, No. (%)		358 (78.7)	117 (74.5)	0.334	233 (80.6)	114 (74.5)	0.172
Sedatives use, No. (%)		224 (49.2)	83 (52.9)	0.488	158 (54.7)	80 (52.3)	0.705

**Non-delirious patients**		**(*N* = 656)**	**(*N* = 246)**		**(*N* = 401)**	**(*N* = 243)**	

Age, mean (SD)		64.9 (14.9)	63.0 (16.0)	0.099	65.0 (15.4)	63.2 (15.9)	0.165
Male, No. (%)		389 (59.3)	155 (63.0)	0.348	239 (59.6)	153 (63.0)	0.445
ICU days, mean (SD)		3.0 (3.2)	3.4 (3.5)	0.140	3.82 (3.5)	3.40 (3.5)	0.136
Type of admission, No. (%)	Medical	153 (23.3)	64 (26.0)	0.450	104 (25.9)	64 (26.3)	0.984
	Surgical	503 (76.7)	182 (74.0)		297 (74.1)	179 (73.7)	
Surgery at admission, No. (%)		466 (71.0)	163 (66.3)	0.190	274 (68.3)	160 (65.8)	0.572
Operation status, No. (%)	None	232 (35.4)	89 (36.2)	0.102	156 (38.9)	88 (36.2)	0.702
	Elective	316 (48.2)	103 (41.9)		167 (41.7)	102 (42.0)	
	Emergency	108 (16.5)	54 (22.0)		78 (19.5)	53 (22.0)	
APACHE II score, mean (SD)		17.3 (7.7)	19.9 (8.1)	<0.001	19.38 (8.1)	19.98 (7.9)	0.359
Intubation use, No. (%)		259 (39.5)	141 (57.3)	<0.001	213 (53.1)	140 (57.6)	0.303
Restraint use, No. (%)		266 (40.5)	146 (59.3)	<0.001	220 (54.9)	145 (59.7)	0.266
Ventilator use, No. (%)		253 (38.6)	140 (56.9)	<0.001	207 (51.6)	139 (57.2)	0.195
Analgesics use, No. (%)		483 (73.6)	182 (74.0)	0.982	304 (75.8)	181 (74.5)	0.777
Sedatives use, No. (%)		155 (23.6)	75 (30.5)	0.043	119 (29.7)	75 (30.9)	0.818

*SD, standard deviation; ICU, intensive care unit; APACHE II, Acute Physiology and Chronic Health Evaluation II; Analgesics (tramadol, oxycodone, pethidine, fentanyl, morphine, acetaminophen, codeine phosphate hydrate, and ibuprofen), Sedatives (midazolam, remifentanil, lorazepam, propofol, fentanyl, and dexmedetomidine hydrochloride).*

After PSM, among a total of 1,606 patients, 559 stayed in the ICU after the visit ban. 289 patients were in the restrictive visiting group and 153 patients were in the non-visiting group among 442 delirious patients. As for the non-delirious group, 401 patients were in the restrictive visiting group and 243 patients were in the non-visiting group among 644 patients. There were no differences in demographic and clinical characteristics between the restrictive visiting group and the non-visiting group in all patients, delirious patients, and non-delirious patients.

### Difference in Clinical Outcomes Between Groups

As presented in Table 2, there was no difference in the incidence of delirium between the restrictive visiting and non-visiting groups (χ^2^ = 1.95, *p* = 0.162). In terms of delirium subtypes, there was a significant difference between the two groups (χ^2^ = 12.88, *p* = 0.002). In detail, the rates of hyperactive and mixed type were higher in patients in the non-visiting group than in the restrictive visiting group, and the hypoactive subtype was lower in patients in the non-visiting group than in the restrictive visiting group. The level of anxiety was significantly higher in patients in the non-visiting group than in the restrictive visiting group (*t* = 2.62, *p* = 0.009).

**TABLE 2:** Matched comparison of clinical outcomes between the restrictive visiting group and non-visiting group.

Variables		Restrictive visiting	Non-visiting	*t* (χ^2^)	*p*
All patients		(*N* = 1047)	(*N* = 559)		
Incidence of delirium No. (%)		323 (30.9)	153 (27.4)	1.95	0.162

**Delirious patients**		**(*N* = 289)**	**(*N* = 153)**		

Delirium subtype No. (%)	Hyperactive	80 (27.7)	54 (35.3)	12.88	0.002
	Hypoactive	151 (52.3)	53 (34.6)		
	Mixed	58 (20.1)	46 (30.1)		

**Non-delirious patients**		**(*N* = 401)**	**(*N* = 243)**		

Anxiety (SD)		52.22 (6.50)	53.46 (4.58)	2.62	0.009

*SD, standard deviation.*

### Risk Factors of Delirium Subtype and High Anxiety

A multinomial logistic regression was implemented to examine differences in risk factors between patients with hyperactive subtype compared with patients with hypoactive subtype, and patients with mixed subtype compared with patients with hypoactive subtype (see Table 3). The analysis was conducted by inputting variables that showed significant differences between the groups as covariates before matching. It was found that no visiting was a risk factor for patients with hyperactive and mixed types. Patients who stayed at the ICU during the visit ban were 1.97 times more likely to have a hyperactive subtype than those who did not (OR = 1.97, 95% CI 1.23–3.16, *p* = 0.005) and were 2.32 times more likely to have a mixed subtype than those who did not (OR = 2.32, 95% CI 1.40–3.84, *p* = 0.001).

**TABLE 3:** A Multinomial logistic regression of risk factors of delirium subtypes.

Subtype^a^	Variables	ß	SE	Wald	OR	*p*	95% CI
Hyperactive	Non-visiting	0.68	0.24	7.98	1.97	0.005	(1.23–3.16)
	Restraint	1.25	0.65	3.68	3.50	0.055	(0.97–12.54)
	Ventilator	0.04	0.26	0.02	1.04	0.879	(0.62–1.74)
	APACHE II	0.02	0.02	1.43	1.02	0.232	(0.99–1.05)
Mixed	Non-visiting	0.84	0.26	10.75	2.32	0.001	(1.40–3.84)
	Restraint	1.41	0.77	3.31	4.08	0.069	(0.90–18.59)
	Ventilator	–0.18	0.28	0.40	0.84	0.529	(0.48–1.45)
	APACHE II	0.02	0.02	1.32	1.02	0.251	(0.99–1.05)

*APACHE II, Acute Physiology and Chronic Health Evaluation II; SE, standard error; OR, odds ratio; 95% CI, 95% confidence interval. ^a^Referent group: Hypoactive.*

A binary logistic regression was performed to examine the differences in risk factors between patients with high anxiety and those with low anxiety levels (see Table 4). The analysis was conducted by inputting variables that showed significant differences between the groups as covariates before matching. It was found that no visiting, restraint use, and intubation use were risk factors for patients with high anxiety levels. Patients who stayed at the ICU during the visit ban were 2.26 times more likely to be in high anxiety group than those who did not (OR = 2.26, 95% CI 1.59–3.21, *p* = 0.000) and patients who used restraints were about 2.33 times more likely to be in the high anxiety group than those who did not (OR = 2.33, 95% CI 1.15–4.73, *p* = 0.019). Conversely, patients who used intubations were 2.44 times more likely to be in the low anxiety group than those who did not (OR = 0.41, 95% CI:0.19–0.90, *p* = 0.026).

**TABLE 4:** A binary logistic regression of risk factors of patients with high anxiety levels.

Variables	ß	SE	Wald	OR	*p*	95% CI
Non-visiting	0.82	0.18	20.71	2.26	0.000	(1.59–3.21)
Restraint	0.85	0.36	5.49	2.33	0.019	(1.15–4.73)
Intubation	–0.88	0.40	4.98	0.41	0.026	(0.19–0.90)
Ventilator	0.36	0.24	2.36	1.44	0.125	(0.90–2.29)
APACHE II	–0.02	0.01	3.47	0.98	0.063	(0.95–1.00)
Sedatives use	0.17	0.23	0.54	1.18	0.463	(0.76–1.84)

*APACHE II, Acute Physiology and Chronic Health Evaluation II; SE, standard error; OR, odds ratio; 95% CI, 95% confidence interval; Sedatives (midazolam, remifentanil, lorazepam, propofol, fentanyl, and dexmedetomidine hydrochloride).*

## Discussion

In the current study, we aimed to examine the difference in the incidence of delirium, subtypes of delirium, and anxiety levels between the restrictive visiting group and non-visiting group in the ICU during COVID-19, and to identify the risk factors of subtypes of delirium and high anxiety group. The results of this study can be summarized as follows: (1) there were no significant differences in the incidence of delirium between the restrictive visiting group and non-visiting group in the ICU; (2) the proportion of hyperactive and mixed subtypes was higher than that of the hypoactive subtype in the no visiting period compared with the restrictive visiting period; (3) anxiety level was higher in the no visiting period than in the restrictive visiting period; (4) no visiting was the only risk factor that caused differences in subtypes of delirium; and (5) no visiting, restraint use, and intubation use were risk factors for patients with high anxiety levels.

In a meta-analysis study, family participation was found to be the most effective in reducing the incidence of delirium among several non-pharmacological interventions (Deng et al., 2020). However, in this study, there was no difference in the incidence of delirium regardless of whether the visit was allowed or not. A potential explanation is that the limited daily visiting hours of restrictive ICU visits might be too short to help reduce the incidence of delirium. The visiting time was limited to approximately 1–2 h a day under the restrictive visiting policy, making it difficult for families to properly interact with patients as soon as they need help or emotional support from the family. Therefore, it was not easy to actively make efforts to prevent delirium. Longer visiting hours under the restrictive visiting policy or flexible visiting policy might have helped to reduce the incidence of delirium in patients relative to those under no visiting policy. However, it should be noted that a prior study also reported a flexible family visitation policy did not lower the incidence of delirium (Rosa et al., 2019) and the result was similar to our study. Considering this, the optimal strategy for preventing delirium in ICU may be linked to the multi-component interventions including flexible family visit, exercise program, and physical environment intervention through the use of earplug and eye masks (Litton et al., 2016; Deng et al., 2020).

This study demonstrated that the proportion of patients with hyperactive or mixed delirium was higher than that of patients with hypoactive subtype in the no visiting period compared to the restrictive visiting period. In addition, in the group of patients who experienced no visits, the proportion of patients with hyperactive and mixed delirium was higher than that in patients with hypoactive delirium, which was not the case in the restrictive visiting group. A previous study found that simulated family presence, only a minute family video message providing calmness and familiarity, was effective in reducing agitation in patients with hyperactive or mixed delirium (Waszynski et al., 2018). Similarly, in the current study, complete blocking of ICU visits changed the course of delirium, increasing patients with hyperactive or mixed delirium characterized by agitation and restlessness. It should be noted that ICU visits can alter the rates of patients with hyperactive or mixed delirium, although they could not lead to a difference in the incidence of delirium. Our results suggest that the delirium subtype might tend to be changed by more effectively responding to patients by family, even under the limited visiting time in the ICU. Future studies should re-examine these findings and reveal specific causes.

The level of anxiety was significantly higher in the no visiting period than in the restrictive visiting period. Furthermore, no visiting was a risk factor for patients with high anxiety levels. This is consistent with previous studies showing that flexible ICU visiting hours are related to reduced severity of anxiety (Fumagalli et al., 2006; Junior et al., 2018). During the visiting period, the family can provide emotional stability by supporting and reassuring the patient despite the limited visiting time. In unfamiliar ICU environments, the patient meets his or her family for a short time and feels comfortable just by the presence of the family. In addition, the family can help patients maintain their emotional stability because they can immediately solve what the patient wants or tell the medical staff what they need instead (Dan et al., 2017). However, the absence of visits might cause the patients to feel alienated and isolated and amplify anxiety by making it difficult to adapt well to the stressful environment. On the other hand, restraint and intubation use were also risk factors for patients with high anxiety levels. This finding is similar to that of prior studies in which many patients who experienced restraint and intubation were associated with feeling fear, tension, and anxiety (Evans and FitzGerald, 2002; Rotondi et al., 2002).

In order to improve the situation in which the absence of visits increases the proportion of patients with hyperactive delirium and the level of anxiety in ICU patients, online video visits can be a viable alternative. Online video visits with smartphone applications provide emotional support through interaction between families while maintaining physical restrictions. Indeed, a previous study reported that online video visits decreased anxiety of ICU patients (Shahdosti et al., 2020). In the study, the intervention group that communicated with the family three times a day through online visits in the morning, evening, and night had a significantly lower anxiety scores 48 h after the intervention than the other group that did not (Shahdosti et al., 2020). This is similar to the study that a family video message reduced agitation in patients with hyperactive or mixed delirium (Waszynski et al., 2018). The results suggest that the family presence itself helps the patients in ICU have psychological comforts regardless of physical proximity. Therefore, during the COVID-19 pandemic, hospitals need to expand practical online video visit services and devise methods other than in-person visits to change the subtypes of delirium and reduce the level of anxiety in ICU patients by maintaining family-centered care.

Our study has several limitations. First, we could not collect detailed data on visitors during the restrictive visiting period. According to the past investigation records and experiences of ICU staff, about 90% of patients had visitors in this ICU, but we could not check the exact visitor rates during the restrictive visiting period in this study. Therefore, we have no information on which patients’ families visited, how long the visit was, and how actively the patient was taken care of during the visits. Second, because this study was conducted as a retrospective study, we could not include variables that independently influence the incidence of delirium, the delirium subtype, and the level of anxiety. Future studies may consider variables known to influence delirium and anxiety, including environmental stimulation (such as noise, light) (Brummel and Girard, 2013), and medical history (such as dementia, alcohol-related disorders, and already existing psychiatric disorders) (Ouimet et al., 2007). Third, delirium subtypes were assessed with both DMSS and RASS, although the two scales have different criteria for classifying delirium subtypes. If only one scale was used for this study, different results could be obtained. Fourth, COVID-19 itself might have an overall effect on patients and families in hospitals. “Corona blue” (depression and anxiety experienced by COVID-19), widespread anxiety in hospitals, and the atmosphere in which patients postpone scheduled surgery and decide to hospitalize themselves more carefully may have made differences in APACHE II scores. Fifth, since it is a retrospective study and it is difficult to consider equivalent doses in a real-world setting due to various drugs, only whether to use analgesics and sedatives were matched. Future studies must consider the doses of analgesics and sedatives. Lastly, we performed this study in a combined medical and surgical ICU in South Korea. Because this was a single center observation, to generalize these results, a multicenter study must be conducted.

To our knowledge, this is the first study to examine the association between no ICU visits during COVID-19 and the incidence of delirium, delirium subtypes, and anxiety level. This finding is significant in that it confirmed the importance of family visits in changing delirium subtypes and alleviating anxiety in ICU patients and provided a foundation for nonpharmacological intervention in the ICU.

## Data Availability Statement

The datasets presented in this article are not readily available because data sharing requires permission from the author’s institution. Requests to access the datasets should be directed to the JO, ojuojuoju@yuhs.ac.

## Ethics Statement

The studies involving human participants were reviewed and approved by Institutional Review Board of Gangnam Severance Hospital. Written informed consent for participation was not required for this study in accordance with the national legislation and the institutional requirements.

## Author Contributions

BK, JC, and JO designed and conceptualized the study. BK and JO conducted the literature search, interpreted the data, and drafted and revised the manuscript. JC, JP, and JO collected data. BK and HK analyzed the data. All authors contributed to the manuscript and approved the submitted version.

## Conflict of Interest

The authors declare that the research was conducted in the absence of any commercial or financial relationships that could be construed as a potential conflict of interest.

## Publisher’s Note

All claims expressed in this article are solely those of the authors and do not necessarily represent those of their affiliated organizations, or those of the publisher, the editors and the reviewers. Any product that may be evaluated in this article, or claim that may be made by its manufacturer, is not guaranteed or endorsed by the publisher.
